# Histopathological and ultrastructure alterations in tongue and parotid tissues of rats in consequence to long-term alcohol intoxication and ameliorative role of 10-dehydrogingerdione

**DOI:** 10.1186/s12903-026-07830-9

**Published:** 2026-02-20

**Authors:** Nadia Fathy Hassabou, Mohamed M. Elseweidy, Amina Fouad Farag

**Affiliations:** 1 Oral and Maxillofacial Pathology, Faculty of Dentistry, October 6 University, Giza, Egypt; 2 Professor of Biochemistry, Faculty of Pharmacy, Zagazig University, Zagazig, Egypt

**Keywords:** Alcohol intoxication, Parotid gland, Tongue tissues, Ki-67, 10-dehydrogingerdione

## Abstract

**Background:**

Alcoholism adversely affects oral health, leading to issues such as dental caries, gingivitis, and oropharyngeal cancers. One notable oral effect is glossitis, which manifested as tongue inflammation. Chronic alcohol consumption can lead to sialadenosis, disrupting salivary gland function.

**Aim:**

This study aimed to demonstrate how alcohol intoxication induces histopathological and ultrastructural degenerative alterations in the tongue and parotid tissues of experimental rats and the therapeutic potential of 10-dehydrogingerdione (10-DHGD).

**Methods:**

A total of 40 albino male rats were categorized equally into four groups for the purpose of investigation. The control group received no medication. For 45 days, the alcohol-treated group was given ethyl alcohol daily orally at a rate of 3.7 g/kg body weight; 10-DHGD-treated group was given a daily orally dosage of 10 mg/kg of freshly prepared extract; and the last group was administered a combination of alcohol and 10-DHGD. Histopathological analysis was performed in all the groups, while Ki-67 was used as an immunohistochemical investigation for any nuclear proliferative activity. Additionally, a transmission electron microscope (TEM) ultrastructural assessment was carried out.

**Results:**

The alcohol-treated group displayed destructive alterations in their tongue and parotid tissues. 10-DHGD intake in combination with alcohol ameliorated such alterations to a certain extent. When comparing the alcohol-treated group’s tissues to those of the other groups under inquiry, an immunohistochemical analysis showed a highly significant decrease in Ki-67 expression. In addition, TEM examination of alcohol examined groups revealed fragmented myofibrils of the muscle of the tongue with dilated RER and swollen mitochondria in both the tongue and parotid samples, which were minimized by concomitant 10-DHGD ingestion.

**Conclusion:**

Long-term alcohol intake provoked intoxication in oral tissues, and 10-DHGD treatment dramatically ameliorated these deteriorating consequences.

**Graphical Abstract:**

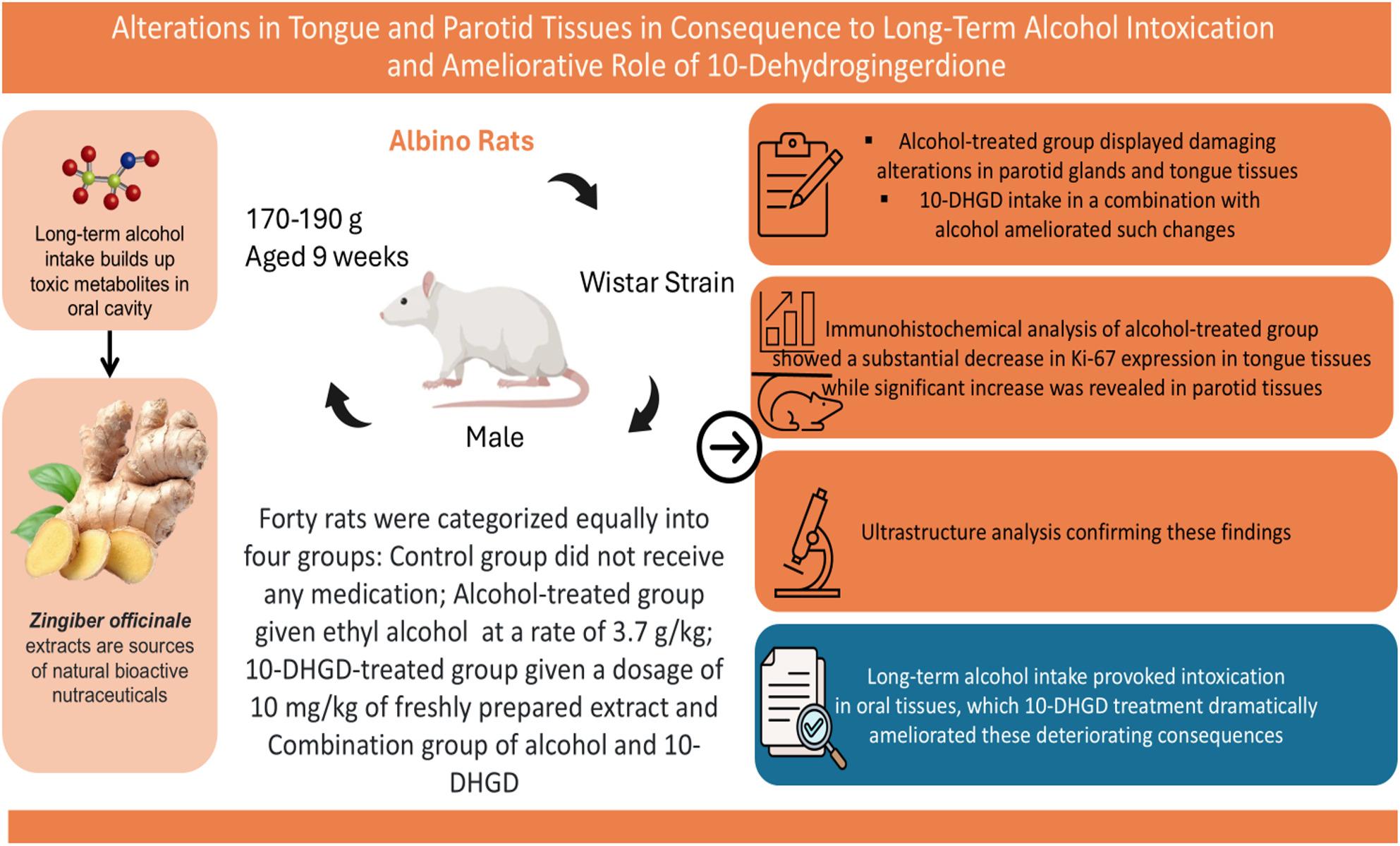

## Highlights

Long-term alcohol intake builds up toxic metabolites in oral cavity

Structural morphology of tongue and parotid tissues can be disrupted by alcoholism

Zingiber officinale extracts are sources of natural bioactive nutraceuticals

10-dehydrogingerdione may provoke potent ameliorative effects

Regenerative response of oral tissues can be modulated by 10-dehydrogingerdione

## Introduction

Alcoholism has many adverse impacts on oral and dental health, extending from dental caries, dental erosions, gingivitis, angular cheilitis, and generalized periodontitis with loss of interdental papillae and alveolar bone resulting in deep pockets to even the development of oropharyngeal cancers [1].

Tongue inflammation or glossitis is one of the most common oral effects of alcoholism, where a painful, smooth tongue with occasional swelling of fungiform papillae is seen in the early stages of glossitis, and an intensely red tongue with atrophy of filiform and fungiform papillae together with altered taste and burning sensation is reported in the later stages [2]. Although such changes resulted from thinning of the maturation layer and associated decreased epithelial thickness, they may be subject to increased risk of cancer development [2, 3].

In medicine, the term “alcohol” typically refers to ethanol (CH_3_CH_2_OH) rather than other alcohols, as this is the active ingredient in alcoholic beverages. Its long-term consumption causes ethanol-induced peripheral neuropathy, which is associated with non-neoplastic, non-inflammatory enlargement and edema of the salivary glands, especially the parotid glands, a condition referred to as sialadenosis [4]. This condition results in disruption in the salivary glands’ metabolism and excretion along with decreased salivary flow (xerostomia) and diminished buffering capacity, and if coupled with poor oral hygiene and a high-carbohydrate diet, it causes a drop in the salivary pH below the critical level, thus increasing the caries index and risk of gingival diseases [5, 6].

Several medicinal plant extracts or their active ingredients are examined for reducing the harmful effects of alcoholism on body tissues and organs. *Zingiber officinale*, family Zingiberaceae, is the source of the natural bioactive nutraceutical 10-dehydrogingerdione (10-DHGD) [7]. It has a promising profile for clinical and medical usage and strong anti-inflammatory, antioxidant, hypolipidemic, and nephroprotective properties [8]. It was proven to repress the NF-κB signaling pathway and inhibit inflammatory and oxidative markers such as Tumor necrosis factor alpha (TNF-α), Nitric oxide (NO), Oxidized Low Density Lipoprotein (Ox-LDL), and Caspase 3, in turn improving inflammation associated with several diseases [9]. 10-DHGD is assumed to offer a novel complementary approach for the improvement of alcoholism-induced adverse effects by its potent inhibitory activities against various significant inflammatory pathways in different body organs and systems [8].

Oral cavity is one of the organs that suffers the side effects of prolonged use of various drugs and lure beverages as alcohol. Although those effects have been investigated on other body organs, only few investigations have shed light on the consequences of prolonged alcoholism on the oral cavity. Accordingly, the need is raised to investigate the deleterious effects of alcohol consumption on the tongue and parotid tissues in addition to the potential ameliorative effect of natural products such as 10-DHGD. To the best of our knowledge, only our earlier published research was found in literature to investigate the probable regenerative effect of 10-DHGD on oral tissues following extended tramadol administration.

## Material and methods

### Animals and study design

Forty male albino rats (Wistar strain) aged 9 weeks and weighing 170–190 g were managed and housed at the Animal Care Facility, Faculty of Pharmacy, Zagazig University, in communal cages (5 animals per cage) with wood shavings as bedding. They were kept under standard conditions (23 ± 2 °C, 60 ± 10% humidity, and a 12:12 h reverse light/dark cycle, lights on at 7:00 AM). A standard chow diet and distilled water were given to rats ad libitum. The rats were examined thoroughly and kept under observation for two weeks by the veterinary staff at the institution before being used as experimental animal models to exclude those that showed signs of any systemic disease. The “Research Ethics Committee at the Faculty of Dentistry, October 6 University (RECO6U/10-2024)” authorized this experimental protocol, which conformed to the “NIH Guide for the Care and Use of Laboratory Animals” and followed the ARRIVE Guidelines Checklist for studies on animals. No private owner approval was needed because the animals were kept in an institutional facility.

All measures were taken to minimize the number of rats used in the current research and their suffering, with sample size estimates calculated based on data from previous studies [8, 9]. Repeated tests using an ANOVA design with a normal distribution assumption found a 0.40 effect size with 95% power and 5% significance (*p* < 0.05). For a significance level of *p* < 0.01, a final sample size of 40 was chosen. The sample size was calculated using G*Power 3.1.9.4.

Following a two-week acclimatization period, rats were randomly assigned to four groups, each consisting of 10 rats (*n* = 10 per group). The classification of rats was conducted as follows: Control group: animals were administered standard chow without any pharmacological interventions; Alcohol-treated group: animals were given alcohol orally once daily for 45 days at a dosage of 3.7 g/kg body weight [8]; 10-DHGD-treated group: animals daily received 10-DHGD at 10 mg/kg body weight orally for 45 days with 2% gum acacia acting as a suspending agent [9, 10]; and combined alcohol and 10-DHGD-treated group: rats given 10 mg/kg body weight of 10-DHGD and 3.7 g/kg body weight of alcohol both orally once daily for 45 days. Oral administration of the studied drugs was performed using a feeding tube (gavage) equipped with a ball-tipped flexible plastic needle, 18 gauge, approximately 75 mm in length. The needle was gently inserted into the esophagus to deliver the compound directly into the stomach, ensuring accurate dosing and minimizing the risk of aspiration or injury [11].

### Drugs and chemicals

10-DHGD was isolated from fresh rhizomes of ginger (“*Zingiber officinale*, family Zingiberaceae”) bought from the herbal market. The extracted material was isolated, identified, and purified in Phytochemistry Department, Faculty of Pharmacy, Zagazig University, Egypt. The extraction and characterization of 10-DHGD was carried out following the method previously described by Choi et al. [12] and Elseweidy et al. [13]. The rhizomes were chopped into fragments and extracted with methanol at room temperature for three days. The methanol extract was then filtered and concentrated under decreased pressure at 45 °C, yielding a dark brown residue. The recovered residue was subsequently suspended in water and blended with n-hexane. The n-hexane layer was separated and concentrated. The residue was put to silica gel for chromatography stages (Merk, silica gel 60, 230–400 mesh, 250 g) with a gradient of n-hexane-ethyl acetate (10:0, 9:1, 8:2, 7:3, 6:4, and 5:5 v/v, 1000 ml each) to get a brownish solid mass. Fraction 5 was separated and applied to GLC-MS analysis of the volatile oil. This was carried out using Clarus 600 Gas Chromatography and Mass Spectrometer Model: Clarus 600 T Mass Spectrometer, USA. Ethanol (≥ 90%) was bought from “Co. For Trading Drugs, Chemicals & Medical Supplies”.

### Body weight measurement

The initial body weight of each rat was recorded on day 0 and then weekly for all rats. Treatment and food were ceased for 12 h at the end of the experiment (day 45), and the animals were again weighed to record the final body weight before euthanasia.

### Euthanasia of animals and collection of tissues

During the experimental time (45 days), no deaths, illnesses, or abnormalities were noted in either the control or the experimental groups. All rats at the end of the study period (day 45) were euthanized using a combination of ketamine hydrochloride (90 mg/kg) and xylazine (10 mg/kg) as an intraperitoneal injection by a well-trained person confirmed with cervical dislocation and subsequent exsanguination carried out according to the recommendations for the euthanasia of experimental animals [14]. Once the corneal and paw withdrawal reflexes were lost, the rats were decapitated to further confirm death, and surgery was conducted to collect the whole tongue and parotid salivary gland tissues of both sides.

The whole tongue of all experimental groups was dissected from the midline into two halves, where its right half together with the parotid gland of the right side was prepared for light microscopic (LM) examination and the left half together with the left gland for transmission electron microscopic (TEM) examination. All glands were weighed after removal by analytical balance (“FA 2104 N, Electronic Balance Bioprecisa, Shanghai, China”), and the relative glandular weight was calculated (gland weight×100/final body weight).

### Light microscopic (LM) examination

Half of the tongue and right parotid glands were immediately fixed in 10% neutral buffered formalin for 48 h, then washed under running tap water, dehydrated in ascending grades of alcohol, cleared in xylene and embedded in paraffin wax. 4 μm thick sections were cut and mounted on regular glass slides for subsequent histopathological analysis using H&E staining protocol [15]. One slide was stained with H&E for routine histomorphometric analysis. Two slides were prepared with immunohistochemical evaluation of proliferative activity following treatment via placing them on a positively charged slide (Optiplus, Biogenex, USA) and immunostained using the three-step indirect streptavidin method [16] for Monoclonal Mouse Anti-Human Ki-67 Antigen (MIB-1), clone M 7240, (DAKO, Denmark). Technical negative controls were obtained by omitting only the primary antibody for the aforementioned marker under identical test conditions. Sections from a lymph node with follicular lymphoid hyperplasia known to be immunoreactive for Ki-67 were used as a positive control (as recommended by the manufacturer) [17, 18].

Using a bright field Leica microscope equipped with a digital camera (BX100) and a computer system for image analysis, two seasoned pathologists independently assessed stained sections. The images were captured at (×200 and ×400) magnification for tongue and salivary tissues, respectively. Then uploaded to the computer system for analysis using Leica Queen Software, Dental Research Laboratory, Faculty of Dentistry, MSA University, Egypt. An analysis was conducted at the same previously mentioned magnifications to determine the Ki-67 immunohistochemical reaction in five microscopic random non-overlapping fields in each specimen that exhibited the most consistent nuclear reaction. The Ki-67 values were presented as the percentage of positive cells in each sample. Based on Igarashi et al., the proportion of proliferating epithelial cells in a sample is calculated by counting a total of 500 cells and then identifying which of those cells are actively dividing, then dividing the number of proliferating cells by the total number of cells counted (500), and finally multiplying by 100 to express the calculated proportion as a percentage [19, 20].

### Preparation of specimens for transmission electron microscopy (TEM)

TEM examination was performed in Electron Microscope Unit, Faculty of Agriculture, Mansoura University, Egypt; on the other tongue halves and left parotid gland tissues following their sectioning at 1 mm^3^ fragments and fixation in 4% paraformaldehyde and 1% glutaraldehyde for two hours. Followed by 1.5 h in 1% osmium tetroxide and phosphate buffer rinsing, the process consisted of three washes in phosphate buffer with a pH of 7.4. After the samples had been fixed, they were dehydrated in ethyl alcohol concentrations that were increasing, then they were cleaned with propylene oxide, and finally they were embedded in epoxy resin. As a means of determining the region that would be subjected to transmission electron microscopy, semithin sections measuring between one and two microns were stained with 1% toluidine blue and examined using light microscopy. Following the cutting of ultrathin sections with an ultramicrotome, the sections were attached to copper grids and stained with uranyl acetate and lead citrate compounds [21].

### Statistical analysis

The gathered data was presented as mean values and measures of variability (mean ± SD) in the statistical analysis that was carried out using the SPSS (Statistical Package for Social Sciences) 28.0 software (IBM, Chicago). The Kolmogorov-Smirnov test was used to evaluate the normal distribution of data. To determine whether or not there were statistically significant differences between the experimental groups, a one-way analysis of variance (ANOVA), followed by Tukey’s HSD post-hoc test was utilized. The statistical significance of the difference was indicated by P values that were less than 0.05. Graphs were constructed by GraphPad Prism^®^ 8 software (GraphPad Software, Inc., Dotmatics, Boston, USA) [22].

## Results

### Body weight and parotid glandular weight

Throughout the experiment, the body weight and parotid mass of each rat were tracked and documented. It was demonstrated that the administration of ethanol (3.7 g/kg body weight), 10-DHGD (10 mg/kg body weight) or a combination of the two on a daily basis for a period of forty-five days did not result in any significant changes to the body weight of the animals (in comparison to the control group). Parotid glandular weight of all experimental rats also demonstrated no significant changes. Comparison between the studied groups in relation to body and glandular weights in each week represented in line graph. As recorded throughout the study; there was no significant difference found during any of the available time periods for analysis when compared to control group, Table 1.

**Table 1:** Comparison between the studied groups in relation to body and parotid gland weights at the end of the study represented in mean ± SD

	GI	GII	GIII	GIV	F	*p*
Body weight/g	187 ± 5.14	181 ± 4.92	185 ± 5.09	183 ± 5.01	0.81136	0.506
Parotid gland weight/mg	35.80 ± 2.3	33.09 ± 1.97	34.89 ± 2.26	34.31 ± 2.01	1.12197	0.369

*SD* Standard Deviation, *F* F-statistic ratio, *p* P-value or probability of observing the data, p ≤ o.o5 indicates non-significant differences

### Histopathological results

#### Control and 10-DHGD-treated groups

**Fig. 1:**
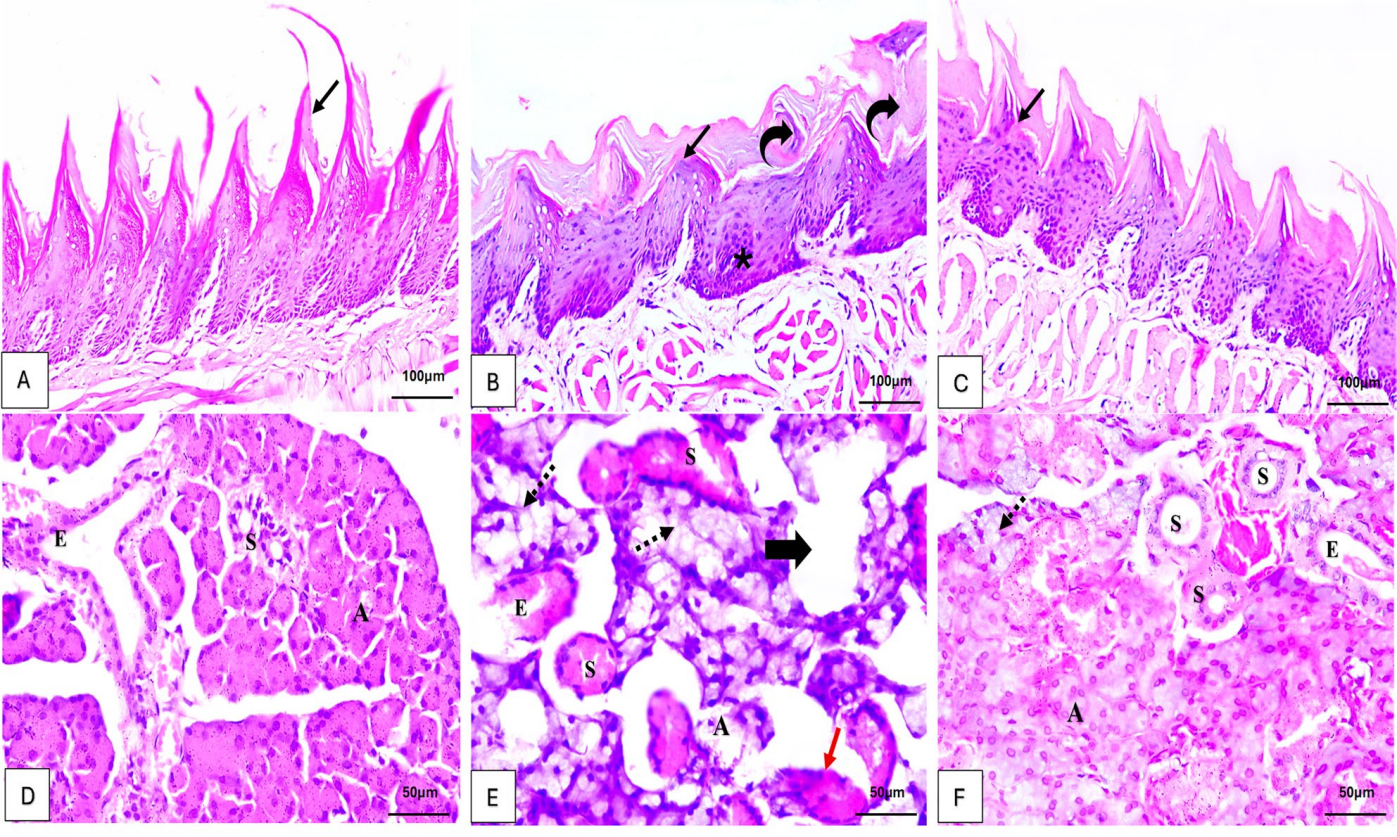
Photomicrographs demonstrating: **A** Tongue tissues of the control group showing filiform papillae with numerous, sharp, and thick keratinized long finger-like projections (arrow). **B** Alcohol-treated group showing poorly defined shallow epithelial ridges with less distinctive conical appearance (arrow), increased stratification of epithelium (*) and keratinization (curved arrows). **C** Combined group demonstrating conical filiform papillae with numerous, sharp, and keratinized long finger-like projections with some less distinctive loss of the conical appearance (arrow), (H&E ×200, Scale Bar = 100 μm). **D** Parotid tissues of the control group showing normal architecture, serous acini ‘A’, excretory ducts ‘E’, and striated duct ‘S’. **E** Alcohol-treated group shows disrupted lobular structure with severely destructed acini ‘A’, cytoplasmic vacuolization (dashed arrows), loss of nuclear basal location (red arrow) and disrupted striated duct ‘S’ and excretory duct ‘E’, wide spacing (thick arrow) between acini is also observed. **F** Combined group showing parotid tissues with minimal acinar destruction, rare cytoplasmic vacuolization (dashed arrow) with distinctive outlines of most of the acini with basal positioning basophilia of the nuclei ‘A’, excretory ducts ‘E’, regenerative striated ducts ‘S’ (H&E ×400, Scale Bar = 50µm)

These groups possessed filiform papillae that were characterized by long projections that resemble fingers on the surface of the dorsal tongue. These papillae were composed of a lamina propria core that was surrounded by thick, stratified squamous epithelium that had normal-shaped epithelial ridges, (Fig. 1A). Normal morphology of the parotid glands represented by acinar and ductal architecture. The serous acini with pyramidal cells were surrounding a small lumen with basal basophilia and rounded nuclei, as well as apical acidophilia, which represent the characteristic histological features of the parotid glands that were found in these groups. The acidophilic reaction of the ducts was higher. Intercalated ducts were lined by low cuboidal to flattened cells that had central nuclei and very little cytoplasm. Striated ducts were lined by columnar cells that were round and single-layered, and they had central nuclei and cytoplasm that was heavily eosinophilic with characteristics of basal striations. Interlobar excretory ducts were lined by stratified cuboidal epithelium, while the main excretory ducts were lined by stratified cuboidal to columnar epithelium before transition to stratified squamous at the ductal opening in the oral cavity, (Fig. 1D).

### Alcohol-treated group

The epithelial ridges of some filiform papillae were shallow and poorly defined, and they lost their conical shape. Aberrant increase in epithelial cell stratification and keratinization was also observed. Special filiform papillae had few, shallow, and poorly defined epithelial ridges and lost their conical shape, (Fig. 1B). In addition, parotid glands showed degraded acini, cytoplasmic vacuolation, losing the basal location of the nuclei and disrupted lobular architecture, (Fig. 1E).

### Combined alcohol and 10-DHGD-treated group

The alcohol and 10-DHGD-treated group had mostly normal filiform papillae, but a few ones lost their conical shape. The epithelial ridge pattern with normalization of keratinization and stratification resembled the control group, with some regions had little, shallow, and poorly defined ridges, (Fig. 1C). Similar to the control group, they had a few areas of vacuolization, cytoplasmic fat droplets, dilated blood vessels and minimal ductal destruction in parotid tissues, (Fig. 1F).

### Immunohistochemical results

**Fig. 2:**
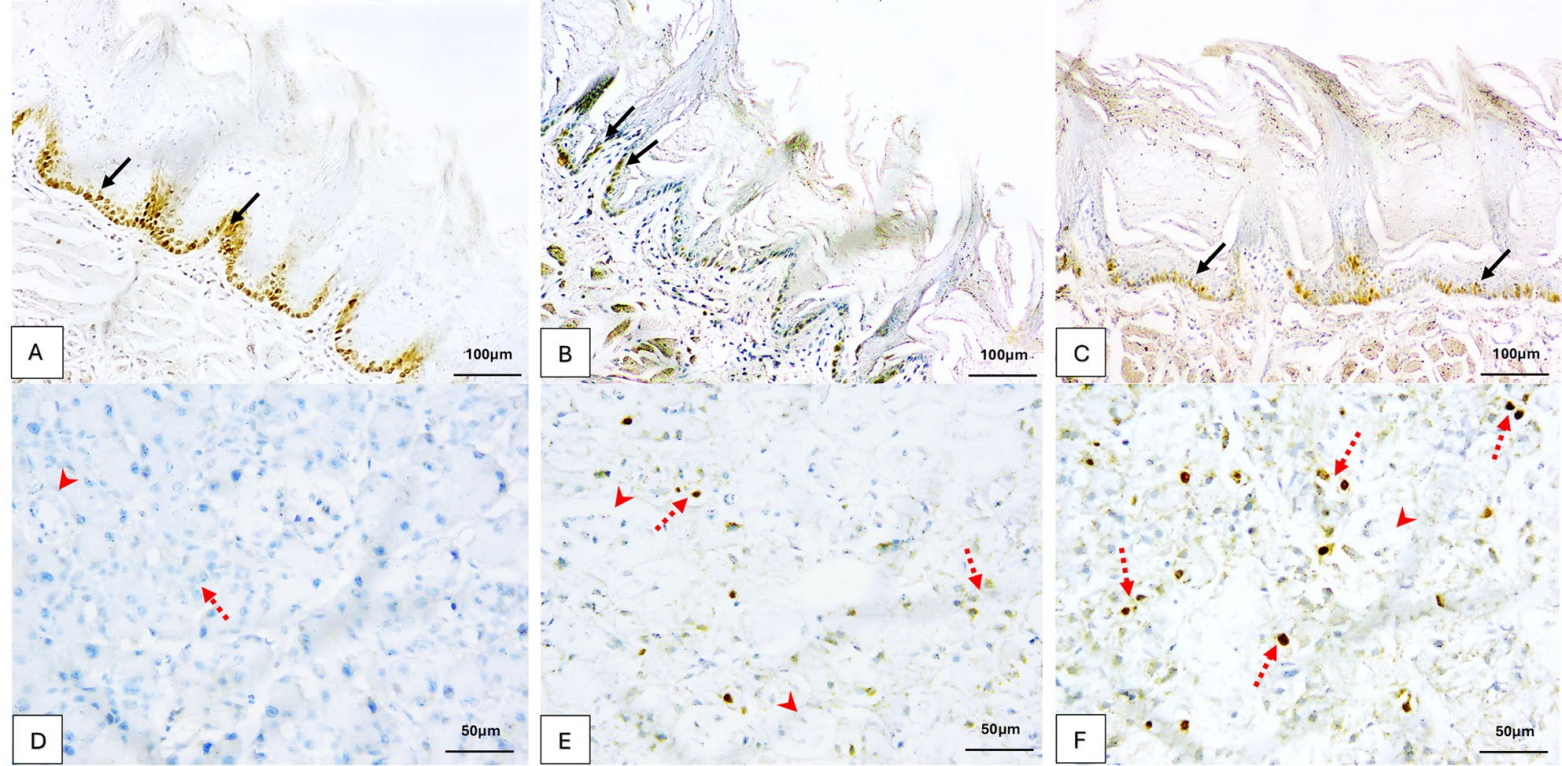
Photomicrographs of Ki-67 immunoreactivity: **A** Normal tongue tissues showing strong brownish nuclear immunoexpression in the basal and suprabasal cells with negative nuclear immunoreaction in the most superficial cells (arrows). **B** Alcohol-treated group demonstrating weak expression (arrows). **C** Moderate brownish nuclear immunoexpression was revealed in the combined alcohol and 10-DHGD-treated group (arrows), (×200, Scale Bar = 100 μm). **D** Parotid tissues of the control group demonstrated a nearly negative Ki-67 nuclear reaction in acinar cells (dashed arrow) and in ductal cells (arrowhead). **E** Alcohol-treated group demonstrating moderate Ki-67 brownish nuclear immunoreactivity in acinar cells (dashed arrows) and negative reaction in ductal cells (arrowheads). **F** Combined group illustrating strong Ki-67 expression in acinar cells (dashed arrows) and weak reaction in ductal cells (arrowhead), (×400, Scale Bar = 50 μm)

The tongue epithelium of the control group and 10-DHGD expressed strong positive nuclear Ki-67 immunoexpression in basal and suprabasal cells and a negative nuclear immunoreaction in superficial cells, (Fig. 2A). The acinar cells in control parotid sections showed negative Ki-67 immunoreactivity, (Fig. 2D).

Weak Ki-67 nuclear immunoreaction was noted in the tongue epithelium (Fig. 2B), but a moderate positive expression was observed in acinar cells (Fig. 2E) within the alcohol-treated group.

It is notable that positivity values of Ki-67 were upraised in the experimental group that received both alcohol and 10-DHGD in contrast to the alcohol group with moderate positive nuclear immunoexpression in tongue epithelium (Fig. 2C) and strong positive nuclear immunoexpression in parotid tissues, (Fig. 2F). The acinar cells may show a mix of granular and homogeneous staining, a predominant granular pattern is a reliable marker of high-turnover, proliferating cells.

**Fig. 3:**
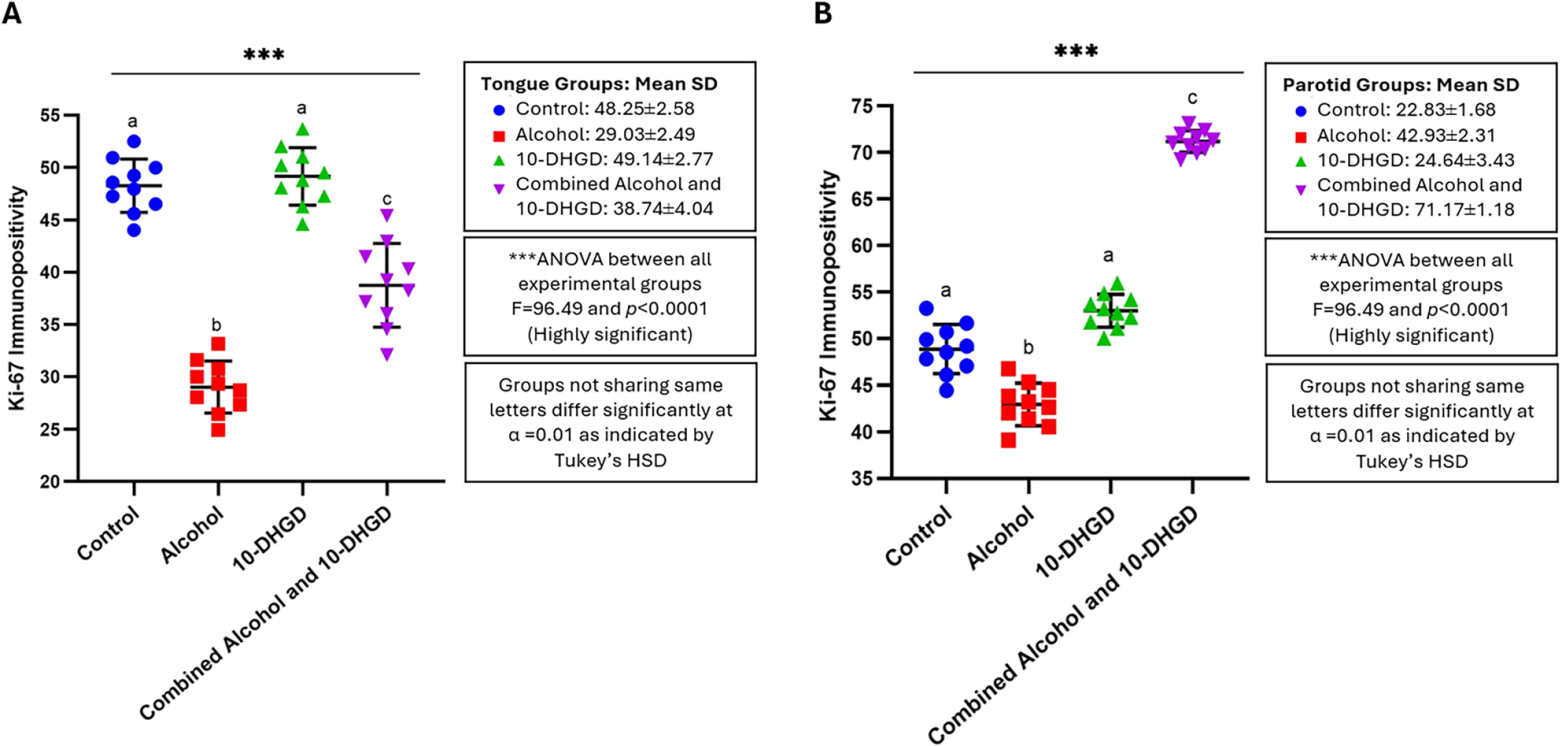
Dotplots with error bars that indicate means and standard deviations (Mean ± SD) associated with Ki-67 immunopositivity in all experimental groups (*n* = 10 per group) using one-way ANOVA followed by Tukey’s HSD post-hoc test for tongue (**A**) and parotid tissues (**B**). Animals in the control group represented by blue dots; the alcohol-treated group by red squares; the10-DHGD-treated group by green apex-up triangles; and the combined alcohol and 10-DHGD-treated groups by purple base-up triangles

The analysis reflected by the one-way ANOVA test in both tongue and parotid tissues was deemed to have a highly significant difference with a large effect between all experimental groups in our present investigation (*p* < 0.0001) (Figs. 3A & B). Pairwise comparison following one-way ANOVA using Tukey’s HSD post-hoc test between the control and 10-DHGD-treated groups yielded a non-significant difference for both tongue and parotid tissues (ANOVA/TukeyHSD; *p* = 0.912 and 0.313, respectively) in contrast to the alcohol-treated group, which yielded a highly significant difference from all other groups (Control, 10-DHGD-treated, and the combined alcohol and 10-DHGD-treated groups), and the combined alcohol and 10-DHGD-treated groups that also yielded a highly significant difference from all other groups (Control, Alcohol and 10-DHGD-treated groups) in both tongue and parotid tissues (ANOVA/TukeyHSD; *p* < 0.0001).

### Transmission electron microscopic (TEM) results

#### Group I (control group) and group III (10-DHGD-treated group)

**Fig. 4:**
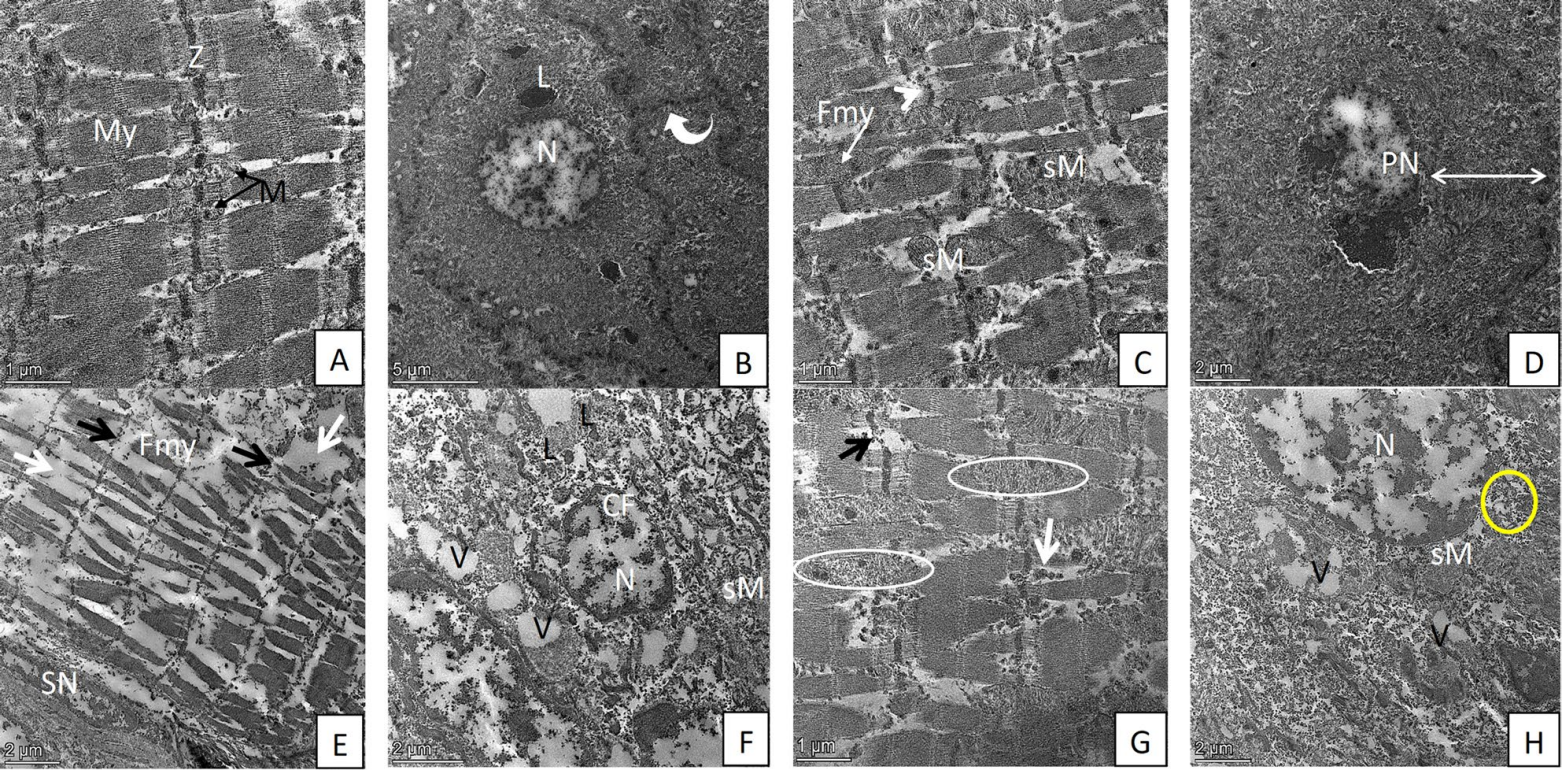
TEM micrograph of tongue tissues from studied groups: **A** control tongue showing longitudinal section of tongue muscles with regular arrangement of myofibril ‘My’ with light and dark band between Z line ‘Z’. Notice, intermyofibril mitochondria ‘M’ besides Z line. **B** A cross section in control tongue epithelium admixed with few electron-dense lysosomes ‘L’ and an euchromatic nucleus ‘N’. Desmosomes (curved arrow) are connecting these epithelial cells. **C** Alcohol-treated group tongue muscles showing irregularly arranged myofibril with fragmentation ‘Fmy’, disorganized or loss of Z line (arrowhead), swollen intermyofibril mitochondria ‘sM’. **D** Cross section of tongue epithelium in the same group with expanded cytoplasmic area (stretched arrow) with pyknotic electron-dense nucleus ‘PN’. **E** Alcohol-treated group muscles showing disruption and lysis of Z band (black arrows) and irregular arrangement of myofibril with diffuse, extensive damage, lysis, shrunken and fragmented myofibril ‘Fmy’, increase intermyofibril space with loss of intermyofibril mitochondria (white arrows), shrunken of sarcolemmal nucleus ‘SN’ with chromatin fragmentation. **F** An epithelial cell of the tongue with numerous vesicles ‘V’, and electron-dense lysosomes ‘L’, swollen mitochondria with loss of cristae ‘sM’, and fragmentation of nuclear chromatin ‘CF’. **G** Longitudinal section of tongue muscle of the combined group showing multifocal, partial fragmentation and lysis of myofibril (circles) with partial loss of Z line (black arrow), numerous intermyofibril small, shrunken, densely stained mitochondria (white arrow). **H** Cross section of tongue epithelium from combined group showing many vesicles ‘V’, swollen mitochondria ‘sM’ with partially disintegrated cristae (yellow circle), Scale Bar = 5 μm (B), 2 μm (D, E, F, H), 1 μm (A, C, G)

**Fig. 5:**
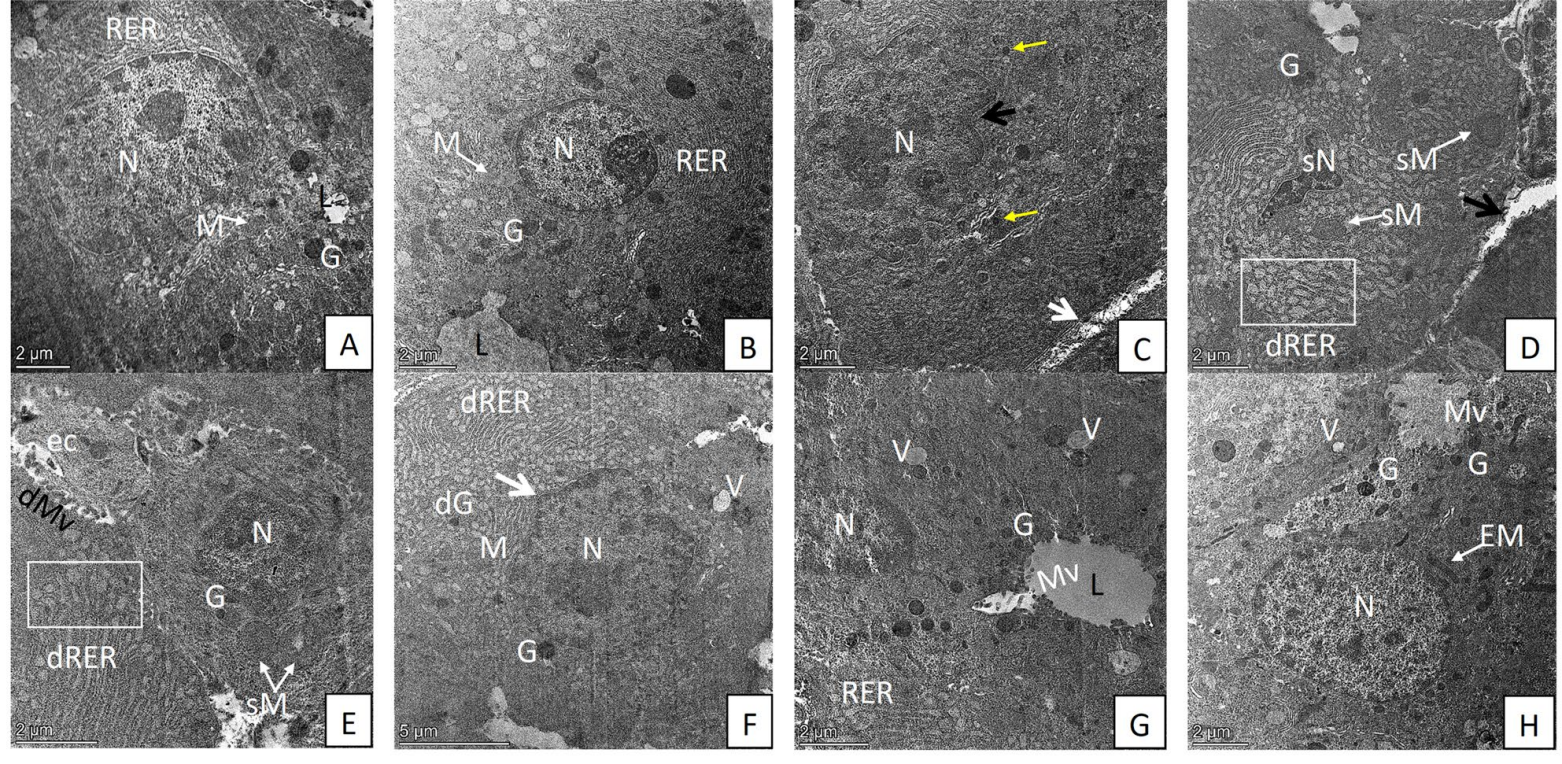
TEM micrograph of parotid gland from different studied groups: **A**, **B** Control parotid tissues showing euchromatic nucleus ‘N’, regular RER ‘RER’, numerous mitochondria ‘M’, luminal surface ‘L’ and numerous apical electron dense secretory granules ‘G’. Alcohol-treated group showing: **C** blebbing of nuclear membrane (black arrow) with few, disintegrated electron dense granules (yellow arrows), widening of the intercellular space (white arrow). **D**, **E** necrosis of acinar cells with small, irregular shrunken nucleus with condensed chromatin ‘sN’, swollen mitochondria ‘sM’, few secretory granules ‘G’, loss of luminal microvillus and widening of the intercellular space (black arrow), exfoliated cellular debris ‘ec’ with desquamated luminal microvilli ‘dMv’, dilated RER (dRER white square), and loss of secretory granules. Combined group showing: **F** partial nuclear membrane blebbing (arrow), a few cytoplasmic vacuoles ‘V’, disintegrated granules ‘dG’ and dilated RER ‘dRER’. **G** Normal an euchromatic nucleus ‘N’, numerous electron-dense apical secretory granules ‘G’, few cytoplasmic vacuoles ‘V’, RER and luminal microvilli ‘Mv’. **H** Acinar cells with nucleus ‘N’, numerous elongated and circular mitochondria ‘EM’, abundant electron-dense apical secretory granules ‘G’, abundant luminal microvilli ‘Mv’ and few cytoplasmic vacuoles ‘V’, Scale Bar= 5 μm (F), 2 μm (A, B, C, D, E, G, H)

TEM examination of tongue muscles of both groups revealed distinctive ultrastructural and highly organized characteristics with long, cylindrical, and parallel myofibrils. Sarcomeres showed a repeating pattern (Fig. 4A). Tongue epithelium showed normal architecture of basal epithelial cells that exhibited numerous desmosomes between the cells with regular smooth nuclear membranes and normal chromatin distribution and regular distribution of cytoplasmic organelles (Fig. 4B). Control parotid tissues showing pyramidal cells with normal euchromatic nuclei, dense regular RER, numerous mitochondria (Figs. 5A & B).

#### Group II (Alcohol-treated group)

TEM examination of tongue tissues revealed irregular arrangement of myofibrils, diffuse and extensive myofibril damage with lysis, shrunken and fragmented myofibrils, and pyknotic electron-dense nuclei with chromatin fragmentation were observed (Figs. 4C & E). Also, an epithelial cell of tongue demonstrated abnormal signs of cytoplasmic degeneration and apoptotic nuclei with fragmented nucleus is seen (Figs. 4D & F).

Ultrastructurally, TEM of salivary gland tissues in group II showed widening of the intercellular space between, necrotic acinar cells with nuclear irregularity and pyknosis, blebbing of nuclear membrane, few secretory granules, and loss of luminal microvilli and secretory granules. Loss of normal architecture of acinar organelles in the form of swollen mitochondria and dilated RER was reported (Figs. 5C, D, E).

#### Group IV (Combined alcohol and 10-DHGD-treated group)

TEM examination revealed some signs of improvement in the ultrastructure alterations that were observed in group II for both tongue (Figs. 4G & H) and parotid tissues (Figs. 5F, G, H) except for a few sections with swollen mitochondria with destroyed cristae and vacuoles (Fig. 4H).

## Discussion

Long-term alcohol abuse represents a serious risk for several diseases that are mainly linked to the induction of microsomal enzymes. This is attributed to the buildup of cytotoxic derivatives like pro-oxidants and acetaldehydes. Alcohol consumption can induce accumulation of hazardous metabolites and ethanol metabolic byproducts such as reactive oxygen species (ROS), affecting in turn the cellular components [23].

In consequence, excess unfolding or misfolded proteins may occur and later accumulate to form hazardous aggregates in the cells’ endoplasmic reticulum (ER). These aggregates trigger the unfolded protein response and other organelle dysfunctions, which ultimately result in inflammation and cell death signaling [24, 25].

It was stated before that ginger extract contains a lot of phenolic and bioactive chemicals; it has shown several medicinal advantages, as it can encourage the growth of new blood vessels that speed up the healing of wounds [26–28]. Taking into consideration the efforts presently being made to find novel, non-toxic medicinal plants as a substitute for medical supplies for the treatment of damaged tissue cells [29]. Oral health is disrupted when the structure and function of the salivary glands are altered, resulting in alterations in the content and flow of saliva and the integrity of the tongue covering [30]. Consequently, the function of 10-DHGD may be clear here as an effective agent regarding oral tissue regeneration in male albino rats in the present study.

The current investigation aimed to examine the degenerative consequences induced by alcohol consumption on tongue and parotid tissues of experimental rats and to illustrate whether 10-DHGD will ameliorate any destructive changes. Based on previous studies carried out by Elnagar et al. [8], Hassabou et al. [10], and Ghosh et al. [31], the selected dose in current research for alcohol was 3.7 g/kg body weight, which is considered a moderate-to-high dose that mimics chronic alcohol consumption in humans, while that for 10-DHGD was 10 mg/kg body weight, which was the most effective concentration for producing optimal therapeutic effects, especially those associated with antioxidant status and histological recovery, with minimal toxicity and other adverse effects in animal models. These dosing regimens were administered orally either alone or in combination, depending on experimental groups, to ensure systemic absorption and to mimic real-life exposure. In addition, they were given on a daily basis for 45 days, which is long enough to trigger oxidative stress, inflammation, and histopathological changes in rat salivary glands and oral tissues.

In the present study, albino rats of the wistar strain were selected to investigate the adverse effects of alcohol on tongue and parotid tissues, as well as the therapeutic potential of 10-DHGD, because of their genetic uniformity, which offers consistent biological responses with reduced variability; their sensitivity to toxins, such as alcohol, which induces oxidative stress and inflammation; and their ease of handling, as they adapt well to lab conditions and long-term studies and provide easy, accurate tissue collection, reproducible biochemical assays, and reliable histological and ultrastructural analyses [32, 33].

Sex differences of experimental rats have been found to be a potential biological variable in alcohol consumption and withdrawal-related behaviors. Male rats were chosen for the current study to avoid hormonal fluctuations that could affect inflammatory, oxidative, apoptotic, and proliferative processes based on studies that revealed differences between males and females in the mechanisms fostering compulsivity as an important facet of alcohol addiction. Moreover, the presence of differences between males and females was reported in the effects of alcohol on the interacting brain systems associated with the three stages of alcohol addiction. Furthermore, tolerance, physical and psychological dependence on, and withdrawal from chronic alcohol consumption have been found to be sex-dependent [34–36]. The experimental parotid gland weight in the rats showed no significant difference when compared to the control group across the analyzed time periods, indicating that the treatment or condition being studied did not significantly affect the gland’s weight.

Our findings indicated significant degenerative alterations in the typical morphology of tongue tissues following alcohol administration and were aligned with the results reported by Altayeb and Salem (2017), who investigated the histological changes of ethanol intake on rat tongues by both LM and scanning EM. They noted that the tongues exhibited irregularly arranged short and long lingual papillae, where certain papillae were slender, exhibiting blunted tips, while others were entirely absent, together with separations of skeletal muscle fibers and vacuolations [37].

Their findings can be explained in a major part by the capability of chronic alcohol consumption to decrease antioxidant defenses while increasing ROS production, which leads to oxidative stress, together with diminished enzyme activity and a reduction in the cellular/extracellular content of non-enzymatic antioxidants. Moreover, ethanol showed the potential to induce carcinogenesis either through disruption of DNA repair mechanisms due to mutations and chromosomal instability associated with cytochrome P450 2E1 (CYP2E1) enzyme and acetaldehyde or through inhibition of DNA methylation, interference with retinoic acid metabolism, and immunosuppressive effects allowing precancerous cells to evade immune surveillance and resulting in tumor development and progression [38, 39].

Abnormal acinar architecture observed in alcohol-treated parotid tissues of studied rats was consistent with that seen in the recent study of Sorkina et al., which demonstrated the long-term effects of alcohol intoxication on rat salivary gland morphology and enzymatic activity [40]. They noted adipose infiltration, stromal edema, and alterations in acinar cells, intercalated ducts, and striated ducts for both submandibular and parotid salivary tissues. They attributed their findings to disorders of metabolism and autonomic innervation of the glands, such as sialadenitis, saliva excretion, and peripheral neuropathy associated with chronic ethanol intoxication that led to changes in the microenvironment in terms of increased numbers of mast cells and reduced antioxidant enzyme activity. Mast cells are the main participants in immune responses to alcohol, as they are very sensitive to the action of various hormones and toxins, including ethanol and lipid peroxidation products, which lead to mast cell activation, enhanced maturation, and intense degranulation [41]. In addition, chronic ethanol intoxication is associated with metabolic imbalances in free radical production leading to oxidative stress and, in turn, reduced levels of antioxidant enzymes such as the nicotinamide adenine dinucleotide phosphate (NADPH) oxidase family, superoxide dismutase (SOD), and glutathione peroxidase (GPx) that damage the gland membranes and internal structures [42–44].

In research conducted by Guan et al., [45] significant adipose infiltration of the parotid glands and sialadenitis was noted in a diabetic patient with a history of chronic alcohol consumption. Chronic alcohol intake may significantly increase the production of ROS, as well as lipid and protein peroxidation, mostly attributed to an interaction with enzymes, membrane lipids, and nucleic acids. Other studies have demonstrated that ethanol and the products of its metabolism can produce ROS and free radicals, which can trigger inflammatory responses that ultimately result in tissue damage [46, 47].

Previous studies that supported our findings on the morphological and ultrastructural levels included those that demonstrated vacuolations and muscle separation among muscle fibers, as well as the accumulation of rounded adipocytes between the muscle fibers. In addition to those studies, they observed a decline in mitochondrial oxidative capacity due to ethanol consumption that may impair the muscle’s capacity to oxidize fat, resulting in lipid accumulation and increased adipocyte formation [37, 48]. These histopathological observations are ultimately related to oxidative stress produced due to alcohol intake [49, 50], meaning ethanol consumption can diminish the levels of antioxidant enzymes [51, 52].

For decades, Ki-67 protein presented itself as a reliable proliferation marker with multiple molecular activities regulating cell cycle progression, such as its contribution to normal cellular distribution of heterochromatin antigens and to the nucleolar association of heterochromatin during interphase, in addition to its role in the formation of the perichromosomal layer (PCL), a ribonucleoprotein sheath coating the condensed chromosomes that prevents aggregation of mitotic chromosomes during mitosis [53].

Therefore, Ki-67 indicates a DNA replication rate associated with cellular proliferation and growth that exhibits a continuous decline during the G0 and G1 phases, followed by a prominent increase from the onset of the S phase until mitotic exit, peaking at G2, representing active phases of the cell cycle, and remaining at diminished levels post cell cycle exit [54]. However, it becomes imperceptible or even absent in senescent or quiescent cells [55, 56]. While Ki-67 is generally considered a nuclear protein, it is not always uniformly distributed. In the G1 phase, it often appears in the perinucleolar region (around the nucleolus, inside the nucleus). In S phase, it often appears as coarser granules throughout the nucleoplasm [57].

Immunohistochemical analyses done in the current study corroborate those performed by Ghoneim et al., Chandrakanta et al., and Takkem et al., where Ki-67 expression differs between salivary gland and tongue tissues, primarily due to variations in tissue type, function, and pathological context. It typically shows low or negligible expression, reflecting the low proliferative activity of long-lived acinar cells of normal salivary glands compared to higher baseline expression in normal tongue epithelium due to its normal constant epithelial turnover. This nuclear expression becomes more profound as the proliferative activity increases and diminishes as it decreases [58–60].

Furthermore, the impact of the difference in tissue architecture between tongue and parotid tissues causes the cytotoxic effects of alcohol exposure to be influenced by different regenerative dynamics and metabolic pathways of these tissues. Damaged tongue epithelial cells are more prone to rapid repair and proliferative compensation following cessation of alcohol intake or regenerative treatment, while the parotid gland’s secretory cells tend to undergo structural degeneration rather than regeneration due to their limited regenerative capability, reflected by lower Ki-67 expression compared to the higher expression presented by tongue cells. Therefore, Ki-67 expression reflects not just damage but also the type of cellular response to injury or harm, and that varies by tissue [61, 62].

The current study showed a significant decrease in Ki-67 positive cells together with atrophic alterations in the both tongue and parotid tissue in the alcoholic group due to ROS suppression of epithelial reproduction by making DNA and RNA non-functional and stopping cells in the S phase as it integrates into them. Consequently, ROS-induced DNA damage and suppression of DNA synthesis affect the metabolism of the progenitor cell, which inhibits mitosis and increases apoptosis. This was expressed as moderate brownish immunoreaction in this group and supported by statistical analysis [63].

10-DHGD is a compound derived from ginger that has shown promising protective effects against alcohol-induced damage in organs like the kidneys [8], liver [64], heart [65], and brain [66]. However, its role as a natural therapeutic option to protect against or counteract the harmful effects of chronic alcohol consumption on oral health, especially the tongue and salivary glands, is still emerging and not widely studied. The histological, immunohistochemical, and ultrastructural findings of current studies were consistent with previously reported studies performed on the possible biological benefits of 10-DHGD due to the high polyphenol content of this flavonoid together with the high concentration of essential amino acids in major protein structures [8–10, 13, 67, 68].

The antioxidant and scavenging properties of 10-DHGD against free radicals are responsible for the principal improvement seen in the group that received a combination of alcohol and 10-DHGD, where the harmful effects of ethanol on the lingual papillae, lingual muscles, and parotid tissues were mitigated and ameliorated by 10-DHGD [8, 69]. It boosts antioxidant defenses by activating the Nrf2 pathway which upregulates protective antioxidant enzymes like SOD, catalase, HO-1, and GPx, which protect tongue cells and parotid glandular cells from alcohol-induced damage by neutralizing ROS associated with oxidative stress [70, 71].

10-DHGD has a proven anti-inflammatory potential, as it can suppress inflammatory markers like pro-inflammatory cytokine TNF-α and Nuclear Factor Kappa Beta (NF-κβ) that result in inactivation of signaling cascades such as Phosphatidylinositol 3-kinase/Protein kinase B (PI3K/Akt) and mitogen-activated protein kinase (MAPK) pathways, which form other inflammatory mediators like IL-1, IL-6, and chemokines and in turn lead to inhibition of leukocyte recruitment, vascular permeability, and tissue damage associated with inflammation [72, 73]. It also inhibits key enzymes involved in the inflammatory process, such as cyclooxygenase-2 (COX-2), responsible for pro-inflammatory prostaglandin formation, and also inhibits lipoxygenase (LOX), which contributes to leukotriene inflammatory mediator production, thereby mitigating inflammation [74]. Thus, 10-DHGD can protect against glossitis and atrophy of tongue coating, glandular inflammation, and shrinkage of salivary tissues, rendering them less vulnerable to the development of dry mouth (xerostomia) and oral infections associated with chronic alcohol consumption.

Furthermore, 10-DHGD may prevent alcohol-triggered apoptosis by modulating caspase-dependent pathways, which are central to programmed cell death. It triggers caspase-3, -8, and -9, reducing their expression or even inhibiting their activation to prevent downstream cell death and preserve cell integrity. It also enhances anti-apoptotic signals of heme oxygenase-1 (HO-1) to protect mitochondria and reduce oxidative stress, which is a major trigger for caspase activation. Additionally, it inhibits upstream inflammatory pathways (TLR4/NF-κB/ERK signaling), which can amplify caspase activity and associated apoptosis [8, 10].

## Conclusion

The present study highlighted the modulating effect brought by the 10-DHGD treatment as reflected by the histological pattern’s improvement. This may suggest its potential to alleviate the pathological changes observed in the tongue and parotid tissues due to alcohol intake. Additional clinical trials are requested to confirm the results of the experimental study done to further clarify the regenerative potential of 10-DHGD during alcohol intake over an adequate amount of time.

### Limitation of the study

The present study aimed mainly to focus on histopathological and ultrastructural alterations due to intoxication in male albino rats. Despite the success demonstrated, there may be certain limitations in using single-sex experimental rats where sex is considered an important biological variable in both preclinical and clinical research associated with the progression and treatment of alcohol addiction. The biochemical analysis of malondialdehyde (MDA) and antioxidant parameter in the tissue support the hypothesis of the toxicity of alcohol on these tissues.

## Data Availability

The datasets used during the current study are available from the corresponding author upon reasonable request. All the data analyzed during this study are included in this published article in the form of tables and figures.

## Abbreviations

10-DHGD: 10-dehydrogingerdione
ABC: Avidin-Biotin Complex ()
CH3CH2OH: Ethanol
COX-2: Cyclooxygenase-2
CYP2E1: Cytochrome P450 2E1
ER: Endoplasmic reticulum
GPx: Glutathione peroxidase
LOX: Lipoxygenase
MDA: Malondialdehyde
MAPK: Mitogen-activated protein kinase
NADPH: Nicotinamide adenine dinucleotide phosphate
NF-κβ: Nuclear Factor Kappa Beta
NO: Nitric oxide
Ox-LDL: Oxidized Low Density Lipoprotein
PCL: Perichromosomal layer
PI3K/Akt: Phosphatidylinositol 3-kinase/Protein kinase B
ROS: Reactive oxygen species
SOD: Superoxide dismutase
TEM: Transmission electron microscope
TNF-α: Tumor necrosis factor alpha
